# Detection of *Schistosoma mansoni* and *Schistosoma haematobium* by Real-Time PCR with High Resolution Melting Analysis

**DOI:** 10.3390/ijms160716085

**Published:** 2015-07-16

**Authors:** Hany Sady, Hesham M. Al-Mekhlafi, Romano Ngui, Wahib M. Atroosh, Ahmed K. Al-Delaimy, Nabil A. Nasr, Salwa Dawaki, Awatif M. Abdulsalam, Init Ithoi, Yvonne A. L. Lim, Kek Heng Chua, Johari Surin

**Affiliations:** 1 Department of Parasitology, Faculty of Medicine, University of Malaya, 50603 Kuala Lumpur, Malaysia; E-Mails: hanysady@yahoo.com (H.S.); romano@um.edu.my (R.N.); wahib_atrosh@yahoo.com (W.M.A.); ahmed_soofi@yahoo.com (A.K.A.); nabilnesr@yahoo.com (N.A.N.); saldawaki@gmail.com (S.D.); fafo_1979@yahoo.com (A.M.A.); init@um.edu.my (I.I.); limailian@um.edu.my (Y.A.L.L.); joharisurin@um.edu.my (J.S.).; 2 Department of Medical Laboratories, Faculty of Medical Sciences, Hodeidah University, 3114 Hodeidah, Yemen; 3 Department of Parasitology, Faculty of Medicine and Health Sciences, Sana’a University, 1247 Sana’a, Yemen; 4 Department of Biomedical Science, Faculty of Medicine, University of Malaya, 50603 Kuala Lumpur, Malaysia

**Keywords:** *Schistosoma mansoni*, *S. haematobium*, real-time PCR, high resolution melting analysis

## Abstract

The present study describes a real-time PCR approach with high resolution melting-curve (HRM) assay developed for the detection and differentiation of *Schistosoma mansoni* and *S. haematobium* in fecal and urine samples collected from rural Yemen. The samples were screened by microscopy and PCR for the *Schistosoma species* infection. A pair of degenerate primers were designed targeting partial regions in the cytochrome oxidase subunit I (*cox1*) gene of *S. mansoni* and *S. haematobium* using real-time PCR-HRM assay. The overall prevalence of schistosomiasis was 31.8%; 23.8% of the participants were infected with *S. haematobium* and 9.3% were infected with *S. mansoni*. With regards to the intensity of infections, 22.1% and 77.9% of *S. haematobium* infections were of heavy and light intensities, respectively. Likewise, 8.1%, 40.5% and 51.4% of *S. mansoni* infections were of heavy, moderate and light intensities, respectively. The melting points were distinctive for *S. mansoni* and *S. haematobium*, categorized by peaks of 76.49 ± 0.25 °C and 75.43 ± 0.26 °C, respectively. HRM analysis showed high detection capability through the amplification of *Schistosoma* DNA with as low as 0.0001 ng/µL. Significant negative correlations were reported between the real-time PCR-HRM cycle threshold (Ct) values and microscopic egg counts for both *S. mansoni* in stool and *S. haematobium* in urine (*p* < 0.01). In conclusion, this closed-tube HRM protocol provides a potentially powerful screening molecular tool for the detection of *S. mansoni* and *S. haematobium*. It is a simple, rapid, accurate, and cost-effective method. Hence, this method is a good alternative approach to probe-based PCR assays.

## 1. Introduction

Schistosomiasis, one of the most common neglected tropical diseases (NTDs), is caused by parasitic trematode worms of the genus *Schistosoma*. Three medically important species known to infect humans are *Schistosoma mansoni* and *S. japonicum*, which cause intestinal schistosomiasis, and *S. haematobium*, which causes urogenital schistosomiasis. Almost 240 million people are infected worldwide and about 700 million people are at risk of this infection [1,2]. *S. haematobium* and *S. mansoni* are endemic in sub-Saharan African, Middle Eastern and North African regions, and South America, while *S. japonicum* is restricted to southern Asia [3].

The optimal effectiveness of schistosomiasis treatment regimens and control programs are usually associated with the accurate and precise diagnosis of the infection. In many low-income countries endemic with schistosomiasis, microscopy remains the gold standard for a fast, easy, and cheap method of identification of *Schistosoma* species eggs in stool and urine samples [4,5,6,7]. However, the capability of microscopy still needs repeated sampling and careful investigation to give greater sensitivity, especially when the infections are light [8]. Several antigen methods for the detection of schistosomiasis such as ELISA and dipstick platforms assays are used for the detection of circulating cathodic (CCA) and anodic (CAA) antigens in blood and urine which are captured by using monoclonal antibodies [9,10,11]. These methods are specific for current infections and provide estimation for infection intensity [12,13,14]. Likewise, schistosomiasis antibody detection methods are highly sensitive, particularly in low transmission areas; however, they cannot differentiate between current and past infections, and blood samples are not easily collected in the endemic areas [8,9,10,11,12,13,14,15].

The increasing number of travellers, immigrants and foreign workers carrying schistosomiasis from endemic areas to non-endemic countries resulted in the development of polymerase chain reaction (PCR) methods which became a crucial, sensitive and specific diagnostic tool, particularly at an early stage of infection or in selection of the optimal treatment [16,17,18,19,20,21]. Hence, the development of PCR technology is a worthy alternative to microscopy-based diagnostic methods, particularly real-time PCR in the identification and differentiation of *S. haematobium* and *S. mansoni* [20,22,23,24,25]. A loop-mediated isothermal amplification (LAMP) assay was found sensitive and rapid for the detection of early *S. japonicum* infection [26]. Likewise, a multiplex real-time PCR method has been evaluated for the detection and quantification of *S. mansoni* and *S. haematobium* infections in an endemic setting and better results in the detection of schistosomiasis than microscopy were reported [20]. Moreover, a genus-specific real-time PCR was found highly sensitive and offered added value in diagnosing imported schistosomiasis among international travellers and migrants [27]. However, many real-time PCR methods use SYBR green dye (non-saturating dye) which may inhibit DNA polymerase when used at high concentrations [28].

High-resolution melting (HRM) analysis is a relatively new post-PCR analysis that allows direct characterization of DNA amplicons. It does not require the multiplex method and has a low reaction cost compared to other molecular methods. The HRM has been used in human clinical studies with limited applications in the diagnosis of parasitic infections, mainly in molecular studies of some protozoa and helminth infections [29,30,31,32,33,34]. However, identification and differentiation between *S. mansoni* and *S. haematobium* using the real-time PCR-HRM assay has not been described. Therefore, we developed a novel HRM protocol using mitochondrial cytochrome oxidase subunit I (*cox1*) gene as a molecular marker for both *S. haematobium* and *S. mansoni* eggs in urine and stool samples respectively collected from Yemen.

## 2. Results

### 2.1. Schistosoma Infection Status

Of the 400 children, 127 (31.8%) were microscopy-positive for schistosomiasis; 95 (23.8%) had urogenital schistosomiasis (eggs were detected in urine samples), 37 (9.3%) had intestinal schistosomiasis and 3.9% of the infected children were co-infected with both *S. mansoni* and *S. haematobium*. Of the 95 *S. haematobium*-positive samples, 21 (21.1%) were of heavy intensity with mean egg per 10 mL (EP10 mL) of 340 (±244) eggs while 74 (77.9%) cases were of light intensity with mean EP10 mL of 17 (±10) eggs. Likewise, 15 (40.5%) and 3 (8.1%) of *S. mansoni* cases were of moderate and heavy intensity with mean eggs per gram stool (EPG) of 212 (±82) and 637 (±93) eggs respectively. Moreover, 19 (51.4%) of the *S. mansoni* cases were light infections with mean EPG of 50 (±23) eggs.

### 2.2. Primers Specificity and Sensitivity

Specificity of the primers was tested with both schistosomes species positive controls, a wide range of human intestinal parasitic DNA and human parasitic infection-negative stools via a single run conventional PCR, and then subjected to the real-time PCR-HRM method. No amplifications were found for the genomic DNA of intestinal helminth and protozoan parasites except amplification and HRM plots for the *Schistosoma*-positive control. Both *Schistosoma* species DNA successfully amplified a predicted 267 bp product on a 2% agarose gel, whereas no bands were seen for the other parasitic DNA. Then positive amplicons were sent for sequencing and confirmed as *S. mansoni* and *S. haematobium* sequences with BLAST then deposited to the Genbank (Genbank ID: 1783061) (accession number: KP294279-KP294306).

**Figure 1:**
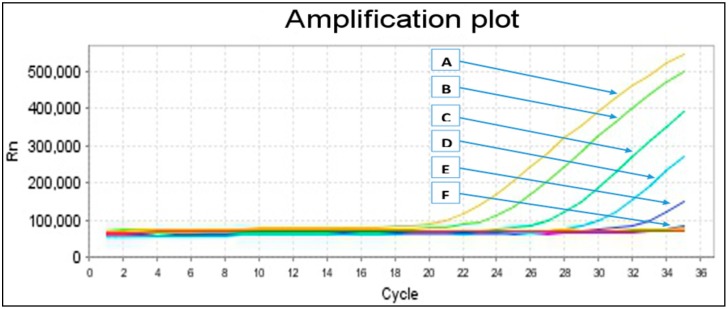
Sensitivity test. A = 10^−1^, B = 10^−2^, C = 10^−3^, D = 10^−4^, E = 10^−5^, F = 10^−6^, 0.0001 ng/µL marked the lowest dilution at which parasite DNA was detected.

Similarly, sensitivity of the primers was determined via a distinct reference DNA control from the positive samples (confirmed by sequencing) to find the lowest DNA concentration that can be detected, as it is known that the copy quantity of parasite genomic DNA existing for each specimen cannot be defined precisely. Evaluation of the sensitivity was done by ten-fold serial dilutions (10^−1^–10^−10^) of 100 ng/µL *Schistosoma* species DNA concentrations that were measured through a Micro UV-Vis fluorescence spectrophotometer (Malcom e-spect, Japan). No amplification was detected either in conventional PCR or real-time PCR-HRM (Figure 1) at a dilution more than 10^−6^ (0.0001 ng/µL), which was marked as the lowest dilution of *Schistosoma* DNA.

**Figure 2:**
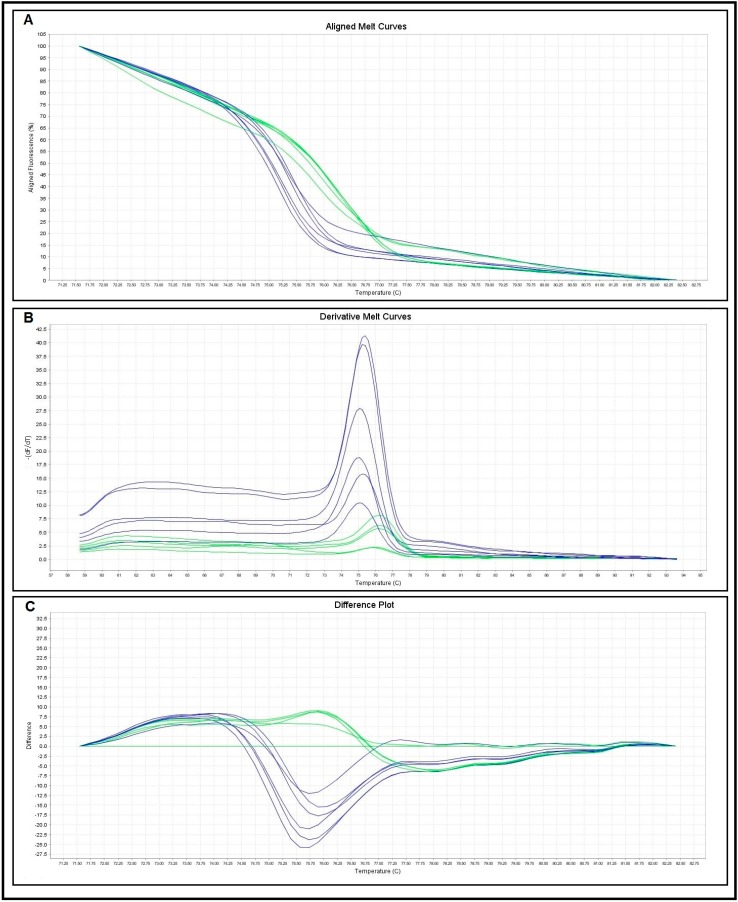
Categorization of *Schistosoma* species based on HRM curve. Representative profiles of the melting curves ((**A**) aligned melt curves; (**B**) derivative melt curves; (**C**) difference plot curves) of COX 1 amplicons for *S. mansoni* (green) and *S. haematobium* (blue), fluorescence is plotted against degrees Celsius (°C).

Three different curves were plotted to evaluate the *cox1* melting characteristics of the two schistosomes species (Figure 2). The normalized fluorescence curves formed through the three melting curves (difference plot, derivative melt curve and aligned melt curve) showed distinctively different plots that simply differentiated the two species. The melting curves of *S. mansoni* and *S. haematobium* were characterized by peaks of 76.49 ± 0.25 and 75.43 ± 0.26 °C respectively (Table 1).

**Table 1:** Melting temperature values of real-time PCR-HRM.

Melting Curve Analysis	No. of Control Samples	Melting Temperatures (Tm) (°C)	
		Range	Mean ± SD
*S. haematobium*	10	75.05–75.82	75.43 ± 0.26
*S. mansoni*	10	76.03–76.79	76.49 ± 0.25

### 2.3. Comparison between Microscopy, Conventional Single Run PCR (Pre-Tested Primers and New Primer) and Real-Time PCR-HRM Assays

Urine and fecal samples were collected from children aged ≤15 years and examined for the detection and differentiation of *Schistosoma* species eggs by three different methods, namely microscopy, conventional PCR and real-time PCR-HRM (Table 2). By microscopy, 95 out of 400 urine samples were found to be positive for *S. haematobium* while 37 fecal samples were positive for *S. mansoni*. After that, pre-tested primers were used and successfully amplified 82% (78 of 95) of the urine samples and 83.8% of the stool samples (31 of 37). Likewise, the new designed primers successfully amplified 91 (95.8%) urine and 34 stool samples (91.9%). With regards to the real-time PCR-HRM, the assay successfully detected all microscopy-positive urine and fecal samples (100%). Interestingly, four and two microscopy-negative urine and fecal samples were found positive for *S. haematobium* and *S. mansoni*, respectively by the real-time PCR-HRM. These extra positive samples were subjected to species confirmation through DNA sequencing followed by BLAST analysis.

**Table 2:** Comparison between microscopy, conventional PCR and real-time PCR-HRM assays for the detection of *S. haematobium* and *S. mansoni* (*n* = 400).

	Microscopy (Reference Method)		Conventional PCR								Real-Time PCR-HRM			
			Pre-Tested Primers				New Primers							
	*S. h.*	*S. m.*	*S. h.*		*S. m.*		*S. h.*		*S. m.*		*S. h.*		*S. m.*	
			+ve	−ve	+ve	−ve	+ve	−ve	+ve	−ve	+ve	−ve	+ve	−ve
**Positive**	95	37	78	17	31	6	91	4	34	3	95	0	37	0
**Negative**	305	363	0	322	0	369	0	309	0	366	4	301	2	361
**Sensitivity**	-	-	82.1%		83.8%		95.8%		91.9%		100.0%		100.0%	
**Specificity**	-	-	100.0%		100.0%		100.0%		100.0%		98.7%		99.45%	
**PPV**	-	-	100.0%		100.0%		100.0%		100.0%		95.9%		94.9%	
**NPV**	-	-	94.9%		98.4%		98.7%		99.2%		100.0%		100.0%	

PPV, positive predictive value; NPV, negative predictive value; +ve, positive; −ve, negative.

Therefore, real-time PCR-HRM increased the overall prevalence rate of schistosomiasis to 33.3% (133 of 400) over the microscopy results (31.8%) and conventional PCR (27.3%). Moreover, the real-time PCR-HRM detected four *S. haematobium* in fecal samples; however urine samples of the relevant participants were also positive. The sensitivity of the real-time PCR-HRM approach was calculated by comparing the results with the reference method (microscopy). The sensitivity of the real-time PCR-HRM was found to be higher than the conventional PCR method used in this study. However, the specificity and positive predictive value for real-time PCR-HRM was lower than the conventional PCR and this was due to the extra positive samples that were detected by real-time PCR-HRM, which were considered as false positives compared to conventional PCR. However, the negative predictive value of real-time PCR-HRM was found to be higher than the conventional PCR method. Moreover, the agreement between the microscopy and real-time PCR-HRM for the detection of *S. mansoni* and *S. haematobium* was statistically significant by Kappa (K = 0.925; *p* < 0.001 and K = 0.887; *p* < 0.001 respectively).

Table 3 shows the real-time PCR-HRM cycle threshold (Ct) values, reflecting parasite-specific DNA loads in samples, per *Schistosoma* egg count (EPG) categories calculated by Kato-Katz and urine filtration techniques for *S. mansoni* and *S. haematobium* respectively. For the PCR-positive samples, the Ct values ranged from 17.4 to 40.0 for *S. mansoni* and from 23.9 to 40.0 for *S. haematobium*. Overall, the results showed significant negative correlation between the Ct values and microscopic egg counts both for *S. mansoni* in stool (*r* = −0.898; *p* < 0.01) and *S. haematobium* in urine (*r* = −0.0935; *p* < 0.01). That said, the median Ct values of *S. mansoni* PCR-HRM-positive samples decreased with increasing egg count categories. Similarly, a decreasing median Ct value of *S. haematobium* PCR-HRM-positive samples was observed with increasing egg count categories.

**Table 3:** Comparison of *S. mansoni*- and *S. haematobium*-specific real-time PCR-HRM according to eggs categorised by number of egg counts.

Egg Count Category	No.	No. (%) Real-Time PCR-HRM +ve	Ct Value	
			IQR	Median
***Schistosoma mansoni***				
Negative	363	2 (0.6)	33.0–36.7	35.3
Light (1–99 EPG)	19	19 (100)	32.0–35.1	33.8
Moderate (100–399 EPG)	15	15 (100)	26.9–31.4	29.8
Heavy (≥400 EPG)	3	3 (100)	23.9–25.3	24.7
***Schistosoma haematobium***				
Negative	305	4 (1.3)	29.2–34.0	31.7
Light (1–50 EP10 mL)	74	74 (100)	25.1–33.9	27.5
Heavy (>50 EP10 mL)	21	21 (100)	20.0–22.3	20.9

EPG, egg count per gram faeces; EP10 mL, egg count per 10 mL urine; +ve, positive; Ct, cycle threshold; IQR, Interquartile range. The median and IQR Ct values were calculated for positive real-time PCR-HRM samples per egg count category.

## 3. Discussion

The present study is the first to describe a novel protocol using the *cox1* gene as a genetic marker by HRM analysis for easy, fast genotyping, species identification and differentiation between *S. mansoni* and *S. haematobium* in human samples. In this protocol, rapid differentiation between *S. mansoni* and *S. haematobium* derived from eggs is based on the unique features of the occurrence curves and melting temperatures for both species. This is in agreement with previous studies done on various parasites such as the detection and differentiation of *Toxoplasma gondii* [29], *Naegleria* spp. [35], *Cryptosporidium* spp. [30], *Dientamoeba fragilis* [36], *Brugia malayi* and *B. pahangi* [31], *Echinococcus* spp. [33], and *Fascioloides magna* [34].

The real-time PCR-HRM method was found to be able to identify sequence variants [37]. Recently, it has been described as a powerful assay for the rapid detection and identification of human hookworms [32], *Brugia malayi*, *Brugia pahangi*, *Dirofilaria immitis* [38], as well as between *S. japonicum* and *S. mekongi* [39]. Moreover, it has been used for the detection of mutations associated with antimalarial drug resistance in *Plasmodium falciparum* genes [40]. In the present study, the assay was designed to amplify a conserved region on the *cox1* gene as it showed sufficient divergence among separate *Schistosoma* species, and a very low level of variation is estimated within isolates collected from the same geographical location [41,42]. Moreover, mitochondrial DNA sequences evolve at a faster rate than nuclear genes and are better suited for discriminating more closely related organisms [42]. Overall, the *cox1* gene is more likely to provide deeper phylogenetic insights than other mitochondrial genes such as cytochrome b because changes in its amino acid sequence occur more slowly than those in any other mitochondrial gene [43,44]. However, further evaluation of the present protocol using clinical samples from different geographical areas is required.

The HRM analysis detects the dissociation (melt) rate of the double stranded DNA (dsDNA) highly fluorescence state using dsDNA-binding dye to the low fluorescence single stranded DNA while increasing temperature during PCR amplification of the target DNA gene region. After that, the high resolution melting stage can be determined by DNA products based on their structure, length, GC content, close neighbor thermo-susceptibility and complementarily. The real-time PCR-HRM method has proven to be a robust assay by providing less contamination through using a closed tube technique; it is more cost effective for each sample; it is easy and fast to read the results via different plotted melt curves for sequences variations; it is less laborious and does not need further amplification as it is performed in nested-PCR; and no fluorescence labelled probes were used for homogeneous genotyping [45,46].

To date, microscopy is still considered as the gold standard method for the detection of parasitic infections, including schistosomiasis, particularly in regions with high endemicity. It has been proven as a cheap and easy method; however, some drawbacks are associated with this method as examination needs to be done within a short time of sample collection, it is tedious, it needs an experienced and well-trained lab technologist and multiple sampling, and long-time screening is necessary to confirm the occurrence with low intensity of infection or the negative results [29,32,47]. Likewise, serology-based assays as alternative methods showed some weaknesses due to the difficulty in differentiating between active and past infection [8,9].

In this study, the real-time PCR-HRM protocol is considered more specific and sensitive in the identification and differentiation of *S. haematobium* and *S. mansoni* only in human samples from a wide range of other intestinal nematode and protozoa control, as well as highly sensitive via its ability to detect DNA as low as 0.0001 ng/µL. Moreover, conventional PCR using pre-tested primers was found to be less sensitive than other methods used in this study. Overall, the positive and negative predictive values found in the present study demonstrate that the HRM protocol was more accurate and precise (100%) to confirm the true negative results than the true positives. It is important to achieve this in the epidemiological survey situation, particularly in the endemic areas where it is better to have a method that can confirm the true negative results. Moreover, this is also useful for selecting optimal treatment and for drug resistance follow-up.

Interestingly, the real-time PCR-HRM assay used here shows the ability to identify four extra *S. haematobium* as well as two *S. mansoni*-positive specimens from six non-egg excretor participants (negative by microscopy and conventional PCR). Hence, the overall prevalence was increased and this may have implications for the control of schistosomiasis in endemic areas. These results confirm previous findings that real-time PCR is highly sensitive for the diagnosis of urogenital schistosomiasis [20]. Hence, the real-time PCR-HRM can be of value on urine and stool samples of suspected patients when no eggs can be detected by microscopy, especially as sampling is not invasive. In the same vein, four *S. haematobium* infections were detected in stool samples (corresponding urine samples were microscopy-positive). Similarly, a recent study reported positive DNA of *S.* *haematobium* eggs in stool samples by using real-time PCR targeting the Dra1 sequence [48]. The detection of *S. haematobium* eggs in faeces may be related to the atypical position of the worm in the rectal or colon wall, as described by earlier reports [49,50]. The present study had to rely on a single fecal/urine sample instead of the ideal three consecutive samples, because of a limitation of resources and the cultural belief of some people against giving their fecal samples. Thus, the prevalence rate of schistosomiasis is likely to be underestimated due to the variation in egg excretion over hours and days. However, this might have no effect on the comparison between the techniques.

## 4. Experimental Section

### 4.1. Sample Collection and Examination

A cross sectional study was carried out from January to July 2012 among a cohort of children aged ≤15 years, living in rural communities in Yemen. Overall, 250 households were randomly selected from twenty villages in Taiz, Ibb, Dhamar, Sana’a and Hodiedah provinces. These five provinces are well known as being endemic for schistosomiasis (Yemen National Schistosomiasis Control Program; NSCP). There were 632 children in these 250 households and they agreed to participate in this study. However, only 400 children successfully submitted the required stool and urine specimens together with the completed questionnaire. A map and detailed description of the study area and population has been published previously in Sady *et al.* [51].

Fecal and urine samples were collected separately into clearly labelled 100 mL clean containers with wide mouths and screw-cap lids. The samples were collected between 10 am and 2 pm when maximum eggs excretion has been shown to occur [52]. For *S. mansoni*, 1 g of each fecal sample was examined for eggs using formalin ether sedimentation and Kato-Katz techniques [53,54]. To determine the worm burden, egg counts were taken and recorded as eggs per gram of feces (EPG) for each positive sample and the intensity of infections was graded as heavy (≥400 EPG), moderate (100–399 EPG) or light (1–99 EPG) according to the criteria proposed by the WHO [53].

About 1 g of each stool sample was preserved in 70% ethanol (DNA-friendly) before being refrigerated [55]. The preserved specimens were transferred to the Department of Parasitology, Faculty of Medicine, University of Malaya, Kuala Lumpur and kept refrigerated for molecular processing.

For *S. haematobium*, urine samples were examined for haematuria using a dipstick test (Self-Stick, Chuncheon, Korea), and then 10 mL were filtered using nucleopore membranes and the filtrate was examined for eggs [56]. Moreover, egg counts were taken and recorded as eggs/10 microliters urine, and intensity of infection was graded as heavy (>50 eggs/10 mL urine) or light (1–50 eggs/10 mL urine) [53].

About 1 mL of sediment from each urine sample was preserved in 70% ethanol before being refrigerated. For quality control and the purpose of the study, duplicate slides were prepared for the samples for each diagnostic technique and the slides were read by two different microscopists.

### 4.2. Parasites Control and DNA Samples

DNA was extracted from stool and urine samples that were previously confirmed positive for *S. mansoni* and *S. haematobium* by microscopy and conventional PCR (described in Section 4.5) and then were chosen to optimize the protocol with new primers for HRM real time PCR. The amplicons were then subjected for purification and sequencing and the results were blasted using Basic Local Alignment Search Tool (NCBI-BLAST) (Available online: http://blast.ncbi.nlm.nih.gov). Specificity of this method was assessed using a collection of positive DNA controls for parasites that were extracted from the Yemeni samples diagnosed with multiple parasitic infections (*Ascaris lumbricoides*, *Trichuris trichiura*, hookworm, *Taenia saginata*, *Fasciola hepatica*, *Giardia lamblia*, *Entamoeba histolytica*, *E. Dispar*, and *E. coli*) and those obtained from Department of Parasitology, University of Malaya (*Strongyloides stercoralis*, *Trichostrongylus* spp.; *Cryptosporidium* spp.; *Blastocystis* spp.; and *E. moshkovskii*). In addition, a negative DNA control from the stool specimen of an individual with no history of parasitic infections and confirmed negative by microscopic examination was used. Moreover, sensitivity of this method was determined by using 10-fold serial dilutions of 100 ng/µL DNA positive controls measured by a Micro UV-Vis fluorescence spectrophotometer (Malcom e-spect, Tokyo, Japan).

### 4.3. DNA Extraction

Before DNA extraction, the stool and urine samples were centrifuged at 2000 rpm for 5 min in 2 mL Eppendorf tubes. The stool and urine precipitants were then washed three times with phosphate-buffered saline (PBS) or MilliQ H_2_O and centrifuged at 2000 rpm for 5 min to remove ethanol. Genomic DNA was extracted from urine samples using DNeasy Blood & Tissue Kit (QIAGEN, Hilden, Germany) while QIAamp DNA Stool Mini Kit (QIAGEN) was used for stool samples according to manufacturer instructions. Briefly, 100 µL of the precipitated urine and 200 mg stool samples were suspended in 180 µL buffer ATL, and 2.6 mL buffer ASL respectively, all tubes were then closed tightly with plastic parafilm. Proteinase K was added and incubated at 56 °C for 1 h before extraction steps were continued. The extracted DNA was finally eluted in 70 µL of AE elution buffer for urine and ATE elution buffer for stool samples (both are included in kit). Then, the extracted DNA was kept at −20 °C until use for PCR amplification.

### 4.4. Primers Design and Pre-Amplification

**Figure 3:**
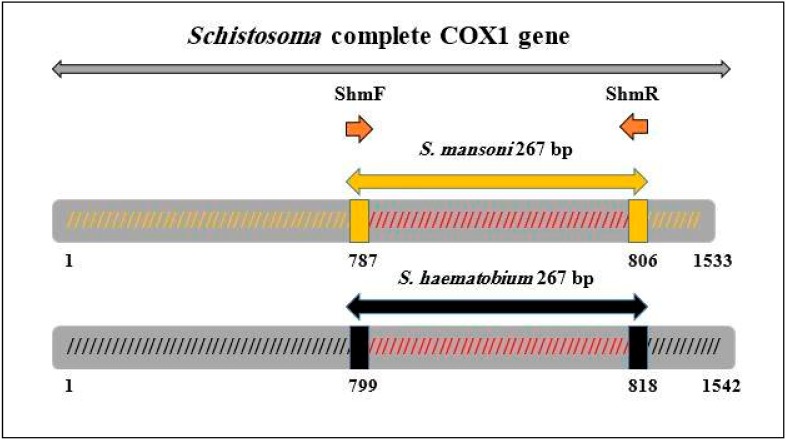
PCR primer sequences and their positions, together with the species PCR fragments in complete *cox1* gene.

A pair of degenerated primers ShmF (Forward 5′-GGATTGATTTGTGCTATGGC-3′) and ShmR (Reverse 5′-CACCGCCWAYCGTAAATAAA-3′) was specifically designed to amplify variable region 267 bp within *cox1* mitochondrial DNA (mtDNA) of *S. mansoni* and *S. haematobium* by real-time PCR coupled with HRM analysis and the conventional PCR as well (Figure 3). This gene was used because mitochondrial DNA is highly abundant in the cell, has highly conserved regions and structures, and its amplification is comparably reliable [57,58,59].

The primers were designed based on two published sequences in the GenBank (accession numbers JQ289741.1 and JQ397397.1). Briefly, the two published sequences were aligned and edited manually to create consensus sequence by BioEdit Sequence Alignment Editor Software 7.2.0 (Available online: http://www.mbio.ncsu.edu) [60]. A single pair of primers was then designed using the sequence analysis and Primer Express software (Applied Biosystems, Inc.; Foster City, CA, USA). Before developing the preferred degenerate primers, confirmation of the created primers was required to make sure that the primers targeted the genomic region of interest by using *in-silico* PCR analysis as described previously [61,62]. This new degenerated primers were ideally designed: length (18–22 bp), high GC ratio content (40%–60%) with a better sensitive detection of *S. mansoni* and *S. haematobium* sequence variants by targeting amplicons length of less than 300 bp within *cox1* gene.

BLAST analysis with primers indicated 100% query coverage and maximum identity with *S. mansoni* and *S. haematobium*. No species other than *Schistosoma* were *in silico* recognized by the primers. Gel electrophoresis obtained a single band of expected length for the amplicon of *S. mansoni* and *S. haematobium* and no signal for the non-template control.

The conventional PCR was used for initial optimization to find the most appropriate typical annealing temperature for the new primers through adjusting series gradient thermal cycler settings (55 to 60 °C) using DNA of *S. haematobium* and *S. mansoni* samples. In addition, non-infected human samples, other intestinal parasitic infection, positive control and distilled water were used. PCR amplification was performed in a total reaction volume of 25 µL comprising 12.5 µL of master mix (HotStarTaq DNA Polymerase, QIAGEN), 400 mM of each primer (ShmF and ShmR) and 2.5 µL of genomic DNA. PCR thermocycling conditions were set at 95 °C for 15 min for initial activation, followed by 35 cycles of 94 °C for 30 s (denaturing), 58 °C for 1 min 30 s (annealing) and 72 °C for 1 min (extension) with a final extension at 72 °C for 7 min. PCR amplification was done in a single PCR reaction using MyCycler thermal cycler (Bio-Rad, Hercules, CA, USA) to produce amplicons of 267 bp for both *S. haematobium* and *S. mansoni* (Supplementary Figure S1A), which was then visualized by fragment separation in 2% agarose gels electrophoresis in 1X TAE buffer and stained with SYBR Safe^®^ DNA gel stain (Invitrogen, Waltham, MA, USA) at 80 V for 45 min and envisioned in a UV transilluminator.

### 4.5. Conventional PCR

To detect schistosome DNA in each urine and stool sample, a multiplex schistosome-specific PCR was performed using the DNA extracted from each sample. The schistosome partial *cox1*mitochondrial DNA (mtDNA) region was amplified using a universal forward primer ShbmF (5′-TTTTTTGGTCATCCTGAGGTGTAT-3′) with three species specific reverse primers, ShR (5′-TGATAATCAATGACCCTGCAATAA-3′) for *S. haeamtobium*, SbR (5′-CACAGGATCAGACAAACGAGTACC-3′) for *S. bovis* and SmR (5′-TGCAGATAAAGCCACCCCTGTG-3′) for *S. mansoni* (referred as pre-tested primers in our study) [63,64]. PCR amplification was performed in 25 μL reactions containing 12.5 μL master mix (QIAGEN Multiplex PCR HotStarTaq DNA Polymerase, Hilden, Germany), 1.6 μM of the universal forward primer (ShbmF), 0.8 μM of each of the three reverse primers (ShR, SbR and SmR) and 2 μL of DNA (~103.7 ng/μL from urine samples and 255.7 ng/μL from fecal samples). PCR cycling conditions were subjected to an initial denaturing step of 95 °C for 3 min, followed by 30 cycles of 94 °C for 30 s, 58 °C for 1 min 30 s and 72 °C for 1 min 30 s, with a final extension of 7 min at 72 °C. Amplicons were visualized and sized (375 bp for *S. mansoni*, 543 bp for *S. haematobium*) on a 2% agarose gel stained with SYBR Safe^®^ DNA gel stain (Invitrogen, Waltham, MA, USA) (Supplementary Figure S1B).

### 4.6. Real-Time PCR-HRM Assay

After optimizing the conditions via conventional PCR, the real-time PCR detection approach was performed in a 7500 Fast real-time PCR system (Applied Biosystems, Inc.; Foster City, CA, USA) in a final volume of 20 µL comprising 1X of MeltDoctor HRM Master Mix (Applied Biosystems, Inc.), 0.6 µM of each primer (ShmF and ShmR), and approximately 5 ng/µL of DNA extract. In addition, DNA positive control and DNA blank (distilled water) was included in each round of PCR. PCR cycling for HRM curve conditions was fixed according to the adjusted protocol (one cycle of enzyme activation at 95 °C for 10 min, followed by amplification for 40 cycles consisting of denaturation at 95 °C for 15 s and annealing at 60 °C for 1 min). Subsequently, the melting step was begun directly in the same real-time PCR machine by increasing the temperature to 95 °C (denaturation) for 10 s, annealing at 58 °C for 1 min, high resolution melting at 95 °C for 15 s and final annealing at 60 °C for 15 s. After PCR amplification, melting curve was produced by observing the changes in the fluorescence of a saturated dye (not inhibit PCR) together with temperature −(dF/dT) which was analyzed by HRM analysis software version 2.0.1 (Applied Biosystems, Inc.) with normalization through choosing the linear area before and after the melting transition. Melting temperature (Tm) was interpolated from the normalized data as the temperature at 50% fluorescence, and the different species from *Schistosoma* were distinguished by plotting the fluorescence difference between normalized melting curves. All *Schistosoma* species specimens in this study were scanned in triplicate to get the melting temperature (Tm) standard deviation (SD).

Two of the randomly selected positive control samples that showed typical melt curve and melting temperature generated by the real-time PCR-HRM were sent for purification. The samples were purified using the Gel/PCR DNA Fragment extraction kit, GENEAID, Taiwan, according to the manufacturer’s instructions, then subjected to DNA sequencing in both directions (forward and reverse primers) with an ABI 3730XL sequencer (Genomics Bioscience & Technology Sdn Bhd, Kuala Lumpur, Malaysia).

### 4.7. Comparison between Microscopy, Conventional PCR and Real-Time PCR-HRM Assays

Overall, urine and stool samples were collected from 400 children aged ≤15 years and examined by microscopy for the detection of *S. haematobium* and *S. mansoni*. Then the samples were examined by single run conventional PCR, and also subjected to the real-time PCR-HRM assay. The specificity, sensitivity, and positive and negative predictive values for those three methods were calculated.

This study was carried out after obtaining approval from the Medical Ethics Committee of the University of Malaya Medical Centre, Malaysia (ref. no: 968.4) as well as from the Yemen Schistosomiasis National Control Project, Ministry of Health and Population and also Hodeidah University, Yemen. Written and signed or thumb-printed informed consents were taken from parents or guardians on behalf of their children before starting the sample collection as previously described in detail [51]. Each participant confirmed to be infected with schistosomiasis was treated with a single dose of 40 mg/kg body weight of praziquantel tablets under observation of the researcher and participating medical officer (Direct Observed Therapy).

### 4.8. Statistical Analysis

Results of microscopy, conventional PCR and real-time PCR-HRM were double-entered by two different researchers into Microsoft Office Excel 2007 spreadsheets. Then, a third researcher cross-checked the two data sets for accuracy and created a single data set. Data analysis was made using IBM SPSS Statistics, version 18.0 (IBM Corporation, New York, NY, USA). For descriptive analysis, the prevalence of infections and detection rates by different methods were expressed in percentages, while median (Interquartile range; IQR) was used to present the real-time PCR-HRM Ct values. Egg counts were found to be not normally distributed, however, there are biological justifications for using the arithmetic mean (±standard deviation, SD) rather than the median or geometric mean to express the egg counts of each *Schistosoma* species [65].

Kappa statistics were used to assess the agreement between microscopy and real-time PCR-HRM. Moreover, Spearman’s non-parametric coefficient (*r*) was used to examine the correlation between the Ct values and *Schistosoma* egg counts. Statistical significance was considered at *p* < 0.05.

## 5. Conclusions

The real-time PCR-HRM protocol described by the present study provides high sensitivity and specificity for the detection and differentiation of *S. mansoni* and *S. haematobium*, and to demonstrate co-infection. In addition, the HRM assay is simple, rapid (does not require electrophoresis), cost effective, and provides results that are easy to analyze, interpret and record. Hence, the HRM assay can be used as an accurate, precise alternative tool to other probe-based genotyping assays, for instance SSCP, RFLP and DNA sequencing for *Schistosoma* parasites and a wide range of other microorganisms in the epidemiological surveys and diagnostic laboratories.

## Supplementary Material

Supplementary materials can be found at http://www.mdpi.com/1422-0067/16/07/16085/s1.
