# Supplementary material for: Detection of Schistosoma mansoni and Schistosoma haematobium by Real-Time PCR with High Resolution Melting Analysis
Source: Int J Mol Sci. 2015 Jul 16;16(7):16085–103. doi: 10.3390/ijms160716085 (PMC4519940; doi:10.3390/ijms160716085)
Supplement: Supplementary file 1 [file ijms-16-16085-s001.pdf]

Supplementary Information

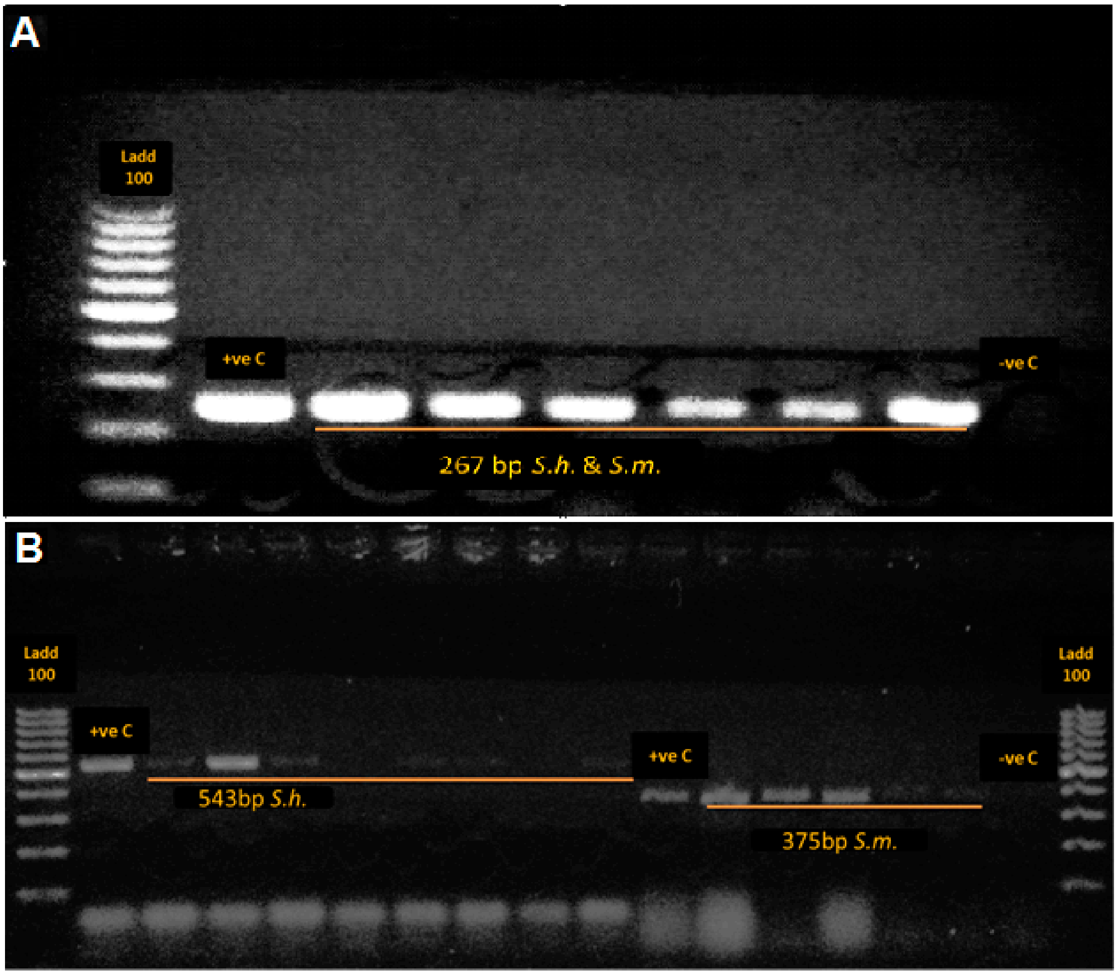

Figure S1. 2% agarose gel electrophoresis of *cox*1 amplicons of *S. haematobium* (*S. h*) and *S. mansoni* (*S. m*) from urine and stool samples, showing the specific PCR profiles (**A**: new primers; **B**: pretested primers). Ladd 100 = 100 bp ladder, +ve C = positive control and −ve C = negative control.
